# Predicting research use in a public health policy environment: results of a logistic regression analysis

**DOI:** 10.1186/s13012-014-0142-8

**Published:** 2014-10-09

**Authors:** Pauline Zardo, Alex Collie

**Affiliations:** School of Population and Global Health, University of Melbourne, Melbourne, Australia; Institute for Safety, Compensation and Recovery Research (ISCRR), Monash University, Melbourne, 3004 Australia; Department of Epidemiology and Preventive Medicine, Monash University, Melbourne, 3004 Australia

**Keywords:** Research use, Predictors, Factors, Policy, Decision-making, Government, Research translation, Intervention

## Abstract

**Background:**

Use of research evidence in public health policy decision-making is affected by a range of contextual factors operating at the individual, organisational and external levels. Context-specific research is needed to target and tailor research translation intervention design and implementation to ensure that factors affecting research in a specific context are addressed. Whilst such research is increasing, there remain relatively few studies that have quantitatively assessed the factors that predict research use in specific public health policy environments.

**Method:**

A quantitative survey was designed and implemented within two public health policy agencies in the Australian state of Victoria. Binary logistic regression analyses were conducted on survey data provided by 372 participants. Univariate logistic regression analyses of 49 factors revealed 26 factors that significantly predicted research use independently. The 26 factors were then tested in a single model and five factors emerged as significant predictors of research over and above all other factors.

**Results:**

The five key factors that significantly predicted research use were the following: relevance of research to day-to-day decision-making, skills for research use, internal prompts for use of research, intention to use research within the next 12 months and the agency for which the individual worked.

**Conclusions:**

These findings suggest that individual- and organisational-level factors are the critical factors to target in the design of interventions aiming to increase research use in this context. In particular, relevance of research and skills for research use would be necessary to target. The likelihood for research use increased 11- and 4-fold for those who rated highly on these factors. This study builds on previous research and contributes to the currently limited number of quantitative studies that examine use of research evidence in a large sample of public health policy and program decision-makers within a specific context. The survey used in this study is likely to be relevant for use in other public health policy contexts.

**Electronic supplementary material:**

The online version of this article (doi:10.1186/s13012-014-0142-8) contains supplementary material, which is available to authorized users.

## Background

Internationally and in Australia, governments are investing in efforts to try and increase evidence-informed public health policy and program decision-making through the establishment of specific research centres and providing funding to support the translation of research [1-3]. There is also an increasing push from government funders to require academics to demonstrate that investments in research deliver a positive impact for the community [4,5]. Whilst this activity demonstrates commitment to driving increased use of research, significant challenges remain. Much is known about the factors that can act as barriers and facilitators to use of research by public health policy and program decision-makers. However, there remains a lack of evidence of how to effectively address these factors in intervention design [1,6,7].

Use of research evidence is context-dependent. The factors that affect research use in one context will not necessarily be the same factors affecting research use in another [8,9]. Context-specific research on factors affecting research use is needed to inform the design and implementation of research translation interventions so that they can be targeted and tailored to the needs of a specific context [10]. Targeting intervention design to the needs of a specific context is expected to enhance effectiveness. Context is affected by individual, organisational and external or societal-level factors [11,12]. To develop a detailed understanding of context, there is a need to understand the factors that operate at these different levels.

A recent study [13] demonstrated that individual factors can significantly affect contextual factors operating at the organisational level. These findings have emphasised the importance of examining factors at the individual, organisational and external level in studies seeking to understand research use in specific public health policy contexts. Studies of the use of research evidence in specific public health policy and program contexts are growing rapidly [14,15]. However, most studies examining the use of research in public health policy environments to date have been qualitative in nature. Fewer studies have quantified the factors that affect research use in specific contexts in large samples of policy and program decision-makers, however, such research is growing [6,16].

This study sought to identify factors that predict use of research evidence in a specific public health policy and program decision-making context using quantitative analysis methods based on survey data.

## Methods

### Context

The two government public health agencies studied, WorkSafe Victoria and the Transport Accident Commission (referred to here on as Agency 1 and Agency 2, respectively) are responsible for workplace and transport injury prevention and rehabilitation compensation for the Australian state of Victoria, which has a population of over 5.5 million people as of September 2012 [17]. These organisations undertake prevention activity and provide financial compensation for the costs of workplace and transport injury and illness rehabilitation treatment and services. They have a very similar mandate but are structured differently and operate differently. In 2009, they partnered with Monash University to establish a research institute dedicated to workplace and transport injury prevention and rehabilitation compensation research.

### Survey development

There are currently no ‘gold standard’ validated surveys assessing the use of research evidence for use with government policy workers [18]. To measure individual-level factors that affect research use and to quantify the type, extent and purpose of use of information, including research evidence, a quantitative survey was developed. A detailed description of the survey is provided in the paper Zardo and Collie, *Use of information in a public health policy environment: Type, frequency and purpose*, submitted 2014. A brief overview of the survey is outlined here.

Survey development was informed by and based on the following: existing literature regarding use of research in health policy decision-making environments, qualitative interviews with employees from Agencies 1 and 2, Michie et al.’s domain framework [19] and questions from a survey developed by Campbell et al. [20].

The survey was comprised of four main parts. Part 1 measured use of information types, frequency of use of information and purpose of use of information (Additional file 1). The information types asked about in the survey included the following: internal data and reports; policy, legislation and legal information; medical and clinical evidence; experience, expertise and advice; information collected online; and academic research evidence. This paper will only focus on the results regarding the use of research evidence. Academic research evidence was defined as follows: peer-reviewed journal articles, reports of academic/scientific research and academic conference abstracts and papers.

Part 2 of the survey was comprised of five questions focused on sources of academic research evidence and engagement in research exchange activities such as attending research forums and collaborating in research projects; the latter reflects Campbell et al.’s [20] study. Part 3 was comprised of 16 questions related to factors that can affect the use of research. Questions were developed in relation to the domains of Michie et al.’s [19] framework to ensure all domains relevant to attitudes and behaviour regarding research use were covered. There were also questions asking about barriers and facilitators to use of research. Part 4 covered demographic questions related to the individual and to their role within their organisation.

Questions for all parts of the survey were focused at the individual level. Questions were framed as follows: ‘How do you use…’, ‘What is your view…’ and ‘In your day-to-day work…’; the emphasis was on the individual’s view and experience about their own behaviour, not that of others. All questions were closed-ended and were compulsory. Answers to all questions were self-reported; therefore, the responses are participants’ perceptions and beliefs regarding their own information use.

A previous paper described results from Part 1 [Zardo and Collie. *Use of information in a public health policy environment: Type, frequency and purpose*, submitted 2014]. This study reports on analyses of data regarding factors affecting the use of research evidence collected in Part 2 and Part 3 of the survey.

#### Survey Testing

A total of eight individuals piloted the survey. Piloting resulted in revision of information-type definitions to provide more detail and greater clarity and description of the survey purpose in the survey introduction and invitation email.

Face validity of the survey was confirmed via feedback from those who piloted the survey, the agencies’ key contacts for this project, as well as via review of the survey by the authors. This survey also covers key concepts addressed in other quantitative surveys in this field [20,21] and expands on these and addresses key concepts outlined in the research translation literature [6,22]. This suggests that content validity is present; however, further review by field experts is required.

To test the reliability of the survey, the split-half method was used, as a measure of internal consistency, to calculate Cronbach’s alpha for 78 valid cases, representing 21% of all cases. Ordinal items only were tested; options for reliability testing of categorical survey items is limited. Cronbach’s alpha for the first random split half was 0.61 and the second 0.56. The Spearman-Brown coefficient for both equal and unequal halves was 0.52. The Guttman split-half coefficient, a conservative measure of split-half reliability, was 0.51. Streiner [23] and others [24] argue that for a survey which is in an early stage of development, a Cronbach’s alpha of 0.50 to 0.60 is acceptable. As there are no ‘gold standard’ surveys published in this field, and as this is the first version of this survey developed and tested, these are positive results. Further analyses and revisions will be undertaken on this survey prior to further use.

### Selection of participants

Potential participants were identified by a review of organisational charts provided by project key contacts. Areas that were involved in the development, implementation or evaluation of policies, program and projects were selected and confirmed by key contacts. All employees from the selected areas (*N* =1,278) were included in the participant pool.

### Recruitment

Potential participants were invited to participate via emails sent by the heads of the selected areas on behalf of the research team. Participants were offered a prize for completing the survey; all participants who fully completed the survey entered the draw.

Invitations were mailed out between 11 and 23 November 2012. Agency 2 sent reminder emails to all selected areas on 10 December 2012. The survey closed on 21 December 2012. Participants self-selected to take part in the study. The program Qualtrics was used to distribute the survey and collect responses.

### Analysis

Survey data was extracted from the Qualtrics programs as a Statistical Package for the Social Sciences version 20 (SPSS) data file and analysed in SPSS. All survey data were categorical. The demographic data were first analysed using crosstabs and chi-square tests to broadly describe the data related to use of research and identify demographics associated with research use. Binary logistic regression was undertaken to determine which factors predict use of academic research evidence. Predictors entered into regression models included all demographic factors and all factors related to research use from Part 2 and Part of 3 of the survey, including the following: barriers and facilitators, research exchange activities, skills, perceived research relevance, difficulty in accessing research, perceived value of research, management support, internal prompts for research use, presentation and communication of research, etc. Ethics approval for the study was received from Monash University Human Research Ethics Committee.

## Results and discussion

Of the 1,278 people invited to participate, 405 fully completed the survey (response rate =31.7%). Thirty-three participants whose main role focus was administration and assistance were excluded, as the research question relates to those who are involved in policy and program development and implementation. This resulted in a sample of 372 participants (Table 1)

**Table 1:** **Research use by demographic factors**

**Demographics**		**Participants**	**Used research**	**Did not use research**	**Univariate logistic regression sig level**
		***N*** **(%)**	***N*** **(%)**	***N*** **(%)**	
All participants (row %)		372 (100)	145 (39.0)	227 (61)	
Agency	1	146 (39.2)	71 (49.0)	75 (33.0)	0.002*
	2	226 (60.8)	74 (51.0)	152 (67.0)	N/A
Role level	Senior manager	15 (4.0)	10 (6.9)	5 (2.2)	0.025*
	Manager	75 (20.2)	33 (22.8)	42 (18.5)	0.215
	Non-manager	282 (75.8)	102 (70.3)	180 (79.3)	N/A
Role focus	Programs and projects	125 (36.6)	64 (44.1)	61 (26.0)	N/A
	Policy and legal	47 (12.6)	22 (15.2)	25 (11.0)	0.608
	Operations	200 (53.8)	59 (40.7)	141 (62.1)	0.000*
Highest level of education	High school/certificate	117 (31.5)	29 (20.0)	88 (38.8)	N/A
	Undergraduate	151 (40.6)	56 (38.6)	95 (41.8)	0.003*
	Postgraduate	104 (27.9)	60 (41.4)	44 (19.4)	0.000*
Gender	Male	121 (32.5)	55 (37.9)	66 (29.1)	1.000
	Female	250 (67.2)	90 (62.1)	160 (70.5)	1.000
	Other	1 (0.3)	0 (0.0)	1 (0.4)	N/A
Age (years)	18–35	163 (43.8)	58 (40.0)	105 (46.3)	N/A
	36–55	187 (50.3)	82 (56.5)	105 (46.3)	0.116
	56+	22 (5.9)	5 (3.5)	17 (7.4)	0.238
Time in agency (years)	0–5	245 (65.9)	88 (60.7)	157 (69.2)	N/A
	6–15	111 (29.8)	51 (35.2)	60 (26.4)	0.073
	16+	16 (4.3)	6 (4.1)	10 (4.4)	0.898
Time in role (years)	0–5	332 (89.2)	132 (91.0)	200 (88.1)	N/A
	6–15	37 (9.9)	11 (7.6)	26 (11.5)	0.238
	16+	3 (0.8)	2 (1.4)	1 (0.4)	0.367
Time in government (years)	0–5	170 (45.7)	58 (40.0)	112 (49.4)	N/A
	6–15	146 (39.2)	60 (41.4)	86 (37.8)	0.202
	16+	56 (15.1)	27 (18.6)	29 (12.8)	0.061

Agency 1 = WorkSafe Victoria; Agency 2 = Transport Accident Commission. All columns are percentages unless otherwise stated. N/A in univariate logistic regression column indicates the comparator variable, for example, Senior manager and Manager use of research was compared to Non-manager use of research.

*Significant at ≤0.05.

.

### Use of academic research evidence

There were 145 participants that indicated that they had used research evidence to inform their decision-making in the last 12 months. Most of those who used research evidence used it monthly (52; 35.9%) or quarterly or less (51; 35.2%). A further 23.4% of participants (34) used research evidence weekly and there were eight participants (5.5%) who used academic research daily.

### Determinants of use of academic research evidence

In total, 49 independent factors were tested using univariate binary logistic regression to identify significant predictors of use of research including demographic factors. Twenty-six of the 49 independent variables significantly predicted the use of research when tested individually.

The 26 factors that significantly predicted research use in univariate analyses included a broad range of factors known to affect research use including the following: skills; access; relevance; value; management encouragement; need to increase research use; time; communication; competing interests; lack of actionable messages; research exchange factors, such as ‘attended a research forum’, ‘participated in a research project’, ‘acted as an advisor on a research project’, ‘invited a researcher to give a perspective on a policy issue’ and individual and organisational demographic factors such as agency, time in agency, time in role and level of education.

These 26 factors were then tested in one block in a multivariate binary logistic regression. Only five factors were found to significantly predict use of research when the effect of all other factors was controlled for in the analysis: skills for use of research, relevance of research, intention to use research, internal prompts for use of research and agency. The five significant predictors of research use were then tested again in one block in a final model.

The final model containing the five predictors was statistically significant (*χ*^2^ (5, *N* =372) =139.28, *p* =0.000), indicating that the model was able to distinguish between participants who did and did not use research. The factors in the model showed standard errors lower than 2.0 indicating that there was no issue with multi-co-linearity [25]. The number of positive cases of use of evidence (*N* =145) is also appropriate for the model, in that there are at least ten positive cases per variable [25].

The model overall explained between 31.2% (Cox and Snell R square) and 42.4% (Nagelkerke R square) of the variance in research use and correctly classified 78.2% of cases. As evident in Table 2 below, all five independent factors made a unique statistically significant contribution to the model. Only the category ‘yes’ related to the factor internal prompts was found not to be significant. Perceived relevance of research was the strongest predictor of use of research, with an odds ratio of 11.87 for ‘relevant’ and an odds ratio of over 5.5 for ‘somewhat relevant’.

**Table 2:** **Significant predictors of research use**

								**95% CI for odds ratio**	
**Predictors**	**Multiple logistic regression**	**B**	**S.E**	**Wald**	**d.f.**	***p***	**Odds ratio**	**Lower**	**Upper**
Skills for use of research	Medium	0.872	0.376	5.369	1	0.020*	2.392	1.144	5.000
	High-very high	1.433	0.399	12.872	1	0.000*	4.192	1.916	9.171
Relevance of research to day-to-day decision-making	Somewhat relevant	1.659	0.772	4.615	1	0.032*	5.253	1.157	23.861
	Relevant	2.474	0.780	10.066	1	0.002*	11.874	2.575	54.758
Intention to use research in the next 12 months	Yes	1.322	0.277	22.785	1	0.000*	3.749	2.179	6.451
Internal prompts for use of research	Not sure	−1.287	0.375	11.784	1	0.001*	0.276	0.132	0.576
	Yes	0.028	0.498	0.003	1	0.955	1.028	0.387	2.731
Agency	Employed by Agency 1	0.794	0.271	8.558	1	0.003*	2.211	1.299	3.763
Constant		3.954	0.786	25.321	1	0.000	0.019		

Agency 1 = WorkSafe Victoria; Agency 2 = Transport Accident Commission.

*Significant at ≤0.05.

This study of 372 decision-makers identified five factors that significantly affect use of academic research evidence in policy and program decision-making in injury prevention and compensation settings in the Australian state of Victoria. These included the following: relevance of research to day-to-day decision-making, skills for using research to inform decision-making, intention to use research in the next 12 months, agency worked with and internal prompts for use of research. This is one of few Australian studies to quantify use of academic research evidence by government policy and program decision-makers on a large scale and to identify specific predictors of research use. These results highlight areas to focus on in the development of research translation interventions in the context studied.

The five significant predictors of research use identified in this context are related to the individual and organisational levels. Other than relevance, the significant predictors identified in this study are factors that could be addressed within the agencies themselves or by the research institute that the agencies jointly fund. It has been argued that research relevance can be enhanced by increased exchange and deliberation between researchers and decision-makers [12,22]. However, it is difficult for researchers to engage in exchange and communication with decision-makers or address factors such as skills through development and delivery of training if decision-makers are not receptive to research or researchers. Internal leadership, senior management level commitment and provision of adequate resources are all factors that have been found to affect decision-makers receptivity to and engagement with research [6,22,26]. As these factors are in the control of the agencies, and not in the direct control of researchers, we suggest that research translation interventions developed for these agencies will need to focus on the policy setting as the primary target for intervention rather than the academic setting.

Whilst it has been identified that researchers can have a critical role to play in increasing and supporting policy and program decision-makers’ use of research [27-29], the findings of this study and systematic reviews of factors affecting research use suggest that in this context there is first a need to build organisational receptivity to research. Organisational receptivity refers to an organisation’s interest in, and capacity for, utilising research evidence to inform decision-making [30-32]. The need to build organisational receptivity for the use of research in Australian government departments generally, and in public health policy decision-making environments specifically, has previously been identified [20,26,31,33,34]. It is expected that supporting the agencies to take ownership and leadership of increasing their organisational receptivity to and capacity for research use will assist in enabling increased engagement and interaction between decision-makers and researchers in this context [22,26,30].

Despite there being many questions in the survey regarding decision-makers’ interaction and exchange with researchers and research forums external to the agency, these were not found to be significant in the final model. When tested individually (in univariate regression analyses), 2 of the 13 options from the section of the survey focused on exchange and communication, ‘sought evidence from a researcher on a regular basis’ and ‘co-authored a publication’, were significant predictors of research use. However, once entered into the model with the other 24 individual predictors they did not remain significant over and above the effect of the five significant variables. This finding is particularly important as increasing opportunities for exchange are frequently recommended as an effective means for driving research use. It is possible that factors such as communication, interaction and engagement may be related to other factors, such as relevance. Factor analysis undertaken as part of future survey refinement will explore this issue. This also highlights that undertaking context-specific research such as this is critical to improving intervention effectiveness, as the factors affecting research use are context-dependent and cannot be assumed [1,22]. It is increasingly argued that any one research translation activity may not be sufficient to increase use of research in policy decision-making [1,35]. Organisations seeking to increase their capacity for use of research will likely need to build research translation platforms or infrastructure that involves a mix of research push, pull, facilitating pull and exchange activities targeted to the needs in their specific context [36,37].

Research relevance has been consistently identified in individual studies and systematic reviews as a critical factor affecting research use [6,38]. Perceived relevance of research was the strongest predictor of research use identified in this study. Participants who viewed research as ‘relevant’ to their role were 11 times more likely to have used research than participants who viewed research as ‘not relevant’. Even participants who viewed research as ‘somewhat relevant’ were five times more likely to use research than those who viewed research as ‘not relevant’. Despite being consistently identified in systematic reviews as a key factor found to affect research use for well over a decade now [7,22,26,38-40], relevance remains a complex issue to understand and address. Notions of relevance held by decision-makers are affected by actual experiences of using research, as well as perceptions and assumptions. Studies by Carol Weiss and others undertaken in the 1980s [41,42] demonstrated that decision-makers perceptions and experiences of the relevance and utility of research are more complex and nuanced than many researchers appreciate.

Weiss and Weiss [42] have also highlighted that any self-reported measures regarding research use are difficult to interpret. This is because it is difficult to determine whether what one says will affect their use of research actually does affect their research use. To explore this issue, Weiss and Weiss examined how individual decision-makers’ self-reported views of research usefulness co-varied with their reports of research utility in relation to practical research case studies. Whilst they did find that decision-makers’ self-reports tended to understate the importance of certain factors expected to affect research usefulness such as ‘applicability of research to existing programs’ or ‘consistency with existing knowledge’, they concluded that ‘decision-makers self- reports about the research characteristics they find useful are for the most part confirmed by their judgments of the usefulness of actual studies’ [42]. It was also interesting to note that researchers and decision-makers assessments of the usefulness and quality of studies were generally comparable.

Weiss and Buculavas [41] showed that decision-makers consider research through three main frames of reference: relevance, truth and utility. Relevance referred to the relevance of the research to decision-maker roles and responsibilities. Truth was composed of two main components, ‘research quality’ and ‘conformity to user experience’ and utility was comprised of the components ‘action orientation’ and ‘challenge to the status quo’. They found that quality was most important when research did not conform to user (decision-maker) experience. However, even when research did conform to user experience, quality remained important. This suggests that contrary to researcher expectations decision-makers remain concerned about quality even when presented with research findings that confirm their expectations. Further and most interestingly, the study showed that decision-makers are interested in and value research that ‘challenges the status quo’ even if they are not able to immediately use it.

This research highlights that perceptions of relevance are complex. Even if decision-makers perceptions of relevance may be enhanced by findings that align with their existing practices and expectations, decision-makers may also expect that research will yield results that challenge the policy ‘status quo’. Kingdon’s research [43] has also highlighted that decision-makers can be open to research and interested in changing direction and views on policy issues but are constrained in their ability to act by a range of contextual factors that are not in their direct control. Weiss’s research [44] has also highlighted that research is often used conceptually, to inform thinking and debate without resulting in immediate action, and argues that this constitutes a valid and valuable use of research. This links with Kingdon’s [43] notion that to create policy change decision-makers must wait for the rare opportunity when the ‘streams’ of ‘policy’, ‘problems’ and ‘politics’ align to create a ‘window of opportunity’ for new policy development or change to occur. This suggests that an initial conceptual use of research by decision-makers can lead to instrumental research use when the right political opportunity or climate presents itself.

In our study, decision-makers were not asked to make an assumption or assertion about what they expected would increase their research use. Decision-makers were asked both if they had used research in the last 12 months and also how relevant they thought research was to their day-to-day work. Their rating of the relevance of research predicted the likelihood of their use of research based on their actual self-reported use of research. Because relevance is a complex concept to interpret, the utility of this finding for informing intervention design would be enhanced by qualitative exploration of what ‘relevance’ means for decision-makers in this specific context.

Increasing skills for use of research has also been identified in systematic reviews as a key strategy for enhancing the use of research [22]. Participants in this study who rated their skills for research use as ‘high’ to ‘very high’ were four times as likely to use research than those who rated their skill as ‘low’, and participants who rated their skill as ‘medium’ were twice as likely to use research. Providing training to improve research skills, including how to access and how to critically appraise research, has been identified as an organisational-level facilitator for use of research [22,36]. It has also been identified as an indicator of organisational receptivity for the use of research [1]. Intervention studies have indicated that provision of training for increasing research skills can increase the ability to appraise research and can also improve perceptions of the potential value of research. To date, however, those studies have not been linked to increases in research use [1].

It is interesting that in this study, participants self-rated skills for research use predicted their use of research when this has not been found previously. The two intervention studies included in Moore’s [1] systematic review sought to increase research skills through the provision of training. The types of training they tested were quite different: one was a half-day training session; the other involved learning modules completed over a 2-year period. Outcomes of both studies focused on whether self-reported ratings of skills for different aspects of research appraisal and use, such as skills for assessing quality, skills in accessing or evidence-seeking behaviour, etc., improved post training completion. As mentioned, increases in use of research were not directly measured. Taylor’s study [45] found improvement in only one aspect of research skills measured which was the ability to appraise systematic review results. Denis’ study [46], which took place over a 2-year period, showed that participants improved on a broader range of research skills.

We did not collect data in our survey on whether or what kind of research skills training participants may have been involved in. Whilst the findings of the studies by Taylor and Denis, as well as our study, suggest that training may be useful in increasing research use, there remains limited knowledge about the types of training and training delivery that are more likely to support increased research use. The findings of Taylor’s and Denis’ [45,46] works suggest that more intensive programs are potentially more valuable than short, once-off training sessions. However, the current body of knowledge regarding research skills is limited and somewhat conflicting, and there is need for further research on the types of training and education that would be useful for increasing research skills and research use.

‘Intention to use research in the next 12 months’ and ‘internal prompts for use of research’ were both significant predictors of research use in this particular decision-making context. Participants who indicated that they intended to use research in the next 12 months were 3.75 times more likely to already be using research. This study also revealed that participants who were unsure about whether there were internal prompts for use of research were not likely to use research. Internal prompts, such as inclusion of sections in project, strategy and business planning templates requiring a review of academic research, reminders in team meetings or weekly newsletters or recognition or accountability for use of research being discussed in performance reviews, could provide a practical means for including research use as part of ‘business as usual’ and support the development of a receptive research climate [36].

That ‘intention to use research in the next 12 months’ predicted research use suggests that encouraging initial use of research by those who have not previously used research may have the potential to increase their continued or future use of research. Over time this could potentially increase the overall number of decision-makers engaging with research. Research by Azjen [47], (best known for the development of the theory of planned behaviour [48]) has shown that both implicit and explicit intentions can predict behaviour. This demonstrates that intentions are powerful predictors of behaviour regardless of whether the intention is made in a general sense such as ‘I will use research in my work’ or specifically, for example ‘I will use research to conduct a literature review to inform the development of project X’. Internal prompts for use of research, such as those outlined above, potentially provide a practical means for both implicitly and explicitly encouraging both ‘first time’ and continued use of research.

The finding that intentions regarding research use predicted use also highlights that decision-makers who are already using research and intend to continue using research may require different intervention activity or targeting than those who have not previously used research. This is supported by research by Dobbins [49] which found that knowledge brokering as an intervention to increase research use was more effective for organisations with low research culture compared to those rated as having a high research culture. Together, these findings highlight that intervention design seeking to increase decision-makers research use must take into account both the organisational context and receptivity, as well as decision-makers individual research capability.

Participants from Agency 1 were more likely to use research evidence than Agency 2. This supports previous research that has found that use of research varies across government agencies [15,21,30]. It is particularly interesting that use can vary significantly between two agencies that have very similar mandates, operate in a homogenous external political environment and have the same primary research provider. This highlights that whilst these agencies are similar, it cannot be assumed that their approach to the use of research and the support needed to drive increased research use are going to be the same. A key difference in the agencies and the survey sample is that Agency 2 employs claims managers and Agency 1 outsources claims management, which is predominately an operational role and is not focused on policy and program development. However, there were participants in the sample from both agencies who identified that their role was focused on operations.

Shine and Bartley [30] have described their very different experiences of working with two very similar policy agencies in very similar contexts. One of the agencies was mandated to use research evidence and the other was not. Interestingly, the agency that was mandated to use research had lower receptivity to research use. They found that the agency that willingly collaborated on research use tended to be positively reinforced by the experience of using research and that conversely their efforts to collaborate with the agency mandated to use research tended to lead to negative reinforcement and receptivity to research remained low. They identified ownership, receptivity and values as key ‘underlying dynamics shaping collaborative efforts’ [30]. They suggest that where it is identified that there is no genuine intention, or no receptivity to accepting or utilising research and that ‘collaborative efforts and public sector reforms are effectively used as a smokescreen to justify ‘business as usual”, research translation efforts may not be appropriate and could even be damaging [30].

Taken together, the analysis by Shine and Bartley [30] and research by Weiss and others [41,42] discussed above reinforces the critical need for researchers to have a deep understanding of the factors affecting and motivating decision-makers use of research. It also highlights that such knowledge is required prior to determining whether a research translation intervention is appropriate, and if it is, to ensure that the intervention is targeted to the needs of the specific decision-makers and organisational context in which the intervention is to be implemented. The findings of this survey and previous research indicate that further research is needed to explore the difference found between the agencies studied and that any research translation intervention strategies will need to be specifically tailored to the needs of each agency.

Whilst the survey has yielded interesting insights, there are limitations. The survey was based on self-report which can be subject to social desirability bias. As the survey was emailed to decision-makers by their head of department, we were careful to ensure that the email invitation and participant information sheet clearly stated that information provided would be kept completely confidential, reporting would be de-identified and that only the research team had access to the data. The email invitation was sent exactly as we crafted it with no altering from the heads of department (the lead researcher was cc’d on all email invitations sent). In all cases, the email was sent by the assistant of the department head on their behalf. It was also made clear that any queries were to be directed to the researchers only and the department heads were not to be contacted nor their assistant and that the email was not to be replied to.

Further this is a new survey, and whilst tested for validity and reliability, further testing and refinement is needed. However, development of the survey was based on existing frameworks specifically designed to inform research on decision-makers use of research evidence developed by leading experts in the field [10,19]. Further, these frameworks were the result of extensive development utilising systematic reviews of research as well as systematic reviews of literature and social and psychological theory regarding motivation and behaviour change that took into account factors at the individual, organisational and environmental level [10,19]. Whilst there are other similar surveys being developed and utilised to measure research use [16,50], there currently remains no gold standard survey available for use in this field.

One of the strengths of this study is that this survey measured use of research in the last 12 months. This provided a practical dependent variable to utilise in the regression analysis. The survey was able to ask decision-makers about factors known to affect research use and examine this in relation to the decision-maker’s actual research use. This meant that decision-makers were not required to make assumptions or state what they expected would increase their use of research; they were rating themselves on factors that are known to affect research use and that was tested against their actual self-reported use of research. Another key strength of this study is that research evidence was specifically defined and defined in relation to other information types. The lack of clear definitions of ‘research’ and evidence has been identified in a recent systematic review as a key issue in the quality of studies conducted to date [26]. The findings of this survey also point to interesting areas for further analysis of data arising from this survey. Further planned analyses include testing whether the predictive factors also predict use of other information types collected in this survey as well as factor analysis to assist further refinement of the survey. Finally, the utility of the findings of the survey in terms of informing research translation intervention design would be substantially advanced by qualitative research that explored the findings further, paying particular attention to the issues raised in this discussion.

## Conclusions

This study has revealed that relevance, skills, intention to use research, internal prompts and agency are significant predictors of use of research in the injury prevention and rehabilitation compensation policy and program context in the Australian state of Victoria. These findings suggest that individual and organisational level factors are the critical factors to target in the design of interventions aiming to increase research use in this context. External factors, such as engagement and interaction with researchers, were not found to affect research use in this study once the effect of other factors were taken into account. The individual and organisational factors that significantly affected research use in this context broadly aligned with previous research in the field. This study builds on previous research and contributes to the currently limited number of quantitative studies to examine the use of research evidence in a large sample of Australian public health policy and program decision-makers.

## Additional file

Additional file 1:
**Use of information survey.** Printout of online survey.
