# Supplementary material for: Predicting research use in a public health policy environment: results of a logistic regression analysis
Source: Implement Sci. 2014 Oct 9;9:142. doi: 10.1186/s13012-014-0142-8 (PMC4212120; doi:10.1186/s13012-014-0142-8)
Supplement: Additional file 1: — Use of information survey. Printout of online survey. [file 13012_2014_142_MOESM1_ESM.pdf]

APPENDIX 3: QUANTITATIVE SURVEY

Introduction

ISCRR Institute for Safety, Compensation and Recovery Research

A joint initiative of WorkSafe Victoria, the TAC and Monash University

Use of Information in Government Decision Making: making research more relevant to everyday practice

This survey is about how you use and how you think about use of information in your day to day work.

This information will help researchers better understand use of information in everyday practice and will inform and improve the way that they work and communicate with people in WorkSafe and the TAC.

***There are 4 Parts to the survey**: Part 1, Use of Information: Part 2, Sources of Information; Part 3, Experience of Use of Information; Part 4, Demographics.*

***This survey is completely confidential.** The researchers are bound by Monash University Ethics to protect your privacy. The researchers will not disclose the names of anyone who completes this survey, at any time*

***PLEASE NOTE**: You will be asked to provide your name. This is only for comparison purposes in the case that you complete this survey again in the future. This information must be kept completely confidential by the researchers and cannot be used for any other purpose, or shared with anyone, for any reason, at any time, including anyone in your organisation.*

Thank you, your contribution is greatly appreciated *Press >> to continue*

A joint initiative of

WorkSafe Victoria   TAC Transport Accident Commission   Monash University

---

Part 1: Use of Information and Evidence

Definitions of Information Types:

For easy reference to these definitions throughout the survey you can open the document attached to the email you received inviting you to participate.

Internal Data and Reports
E.g. Information, data and statistics collected, analysed and reported internally such as claims data, treatment payments data, visits data, information and data collected in Fieldlink, ACCtion, Avanti, program/project/policy management reports etc., any data collected and reported through a data or research unit i.e. BPM or Business Intelligence, Social Research Unit, data collected from Agents, etc.

Policy, Legislation and Legal Information
E.g. The Act, Regulations, or policies and guidelines developed and/or administered by your organisation, or by other government, legal/court decisions, case law, industrial relations law, other legal documents and legal advice etc.

Medical/Clinical Evidence
E.g. Medical certificates, doctors certificates, evidence provided in medical reports and hospital notes, any

documentation provided by a GP, medical specialists, hospitals, pharmacies, etc.

Experience/Expertise/Advice
E.g. Professional experience, expertise, advice and anecdote from people inside or external to your organisation, any information or advice you gain by asking questions and talking to people about your work like your colleagues, your manager, clinical panel members, stakeholders, technical specialists, experts, etc.

Academic Research Evidence
E.g. Peer reviewed journal articles, reports of academic/scientific research, academic conference abstracts and papers.

Information Collected Online
I.e. NOT information/evidence that fits into the other categories. E.g. any other information/evidence you have got from the Internet. Online info can include; documents from your own organisation publicly available online, government documents, other organisations documents and websites, newsletters, any websites/webpages, etc.

Thinking about the last 12 months...

Which of the following types of information have you used in your work?

- ☐ Internal Data and Reports
- ☐ Policy, Legislation and Legal Information
- ☐ Medical/Clinical Evidence
- ☐ Experience/Expertise/Advice
- ☐ Academic Research Evidence
- ☐ Information Collected Online

Thinking about the last 12 months...

How often have you used Internal Data and Reports in your work?

- ○ Daily
- ○ Weekly
- ○ Monthly
- ○ Quarterly
- ○ Yearly

Thinking about the last 12 months...

How often have you used Policy, Legislation and Legal Information in your work?

- ○ Daily
- ○ Weekly
- ○ Monthly
- ○ Quarterly
- ○ Yearly

Thinking about the last 12 months...

How often have you used Medical/Clinical Evidence in your work?

- Daily
- Weekly
- Monthly
- Quarterly
- Yearly

Thinking about the last 12 months...

How often have you used Experience/Expertise/Advice in your work?

- Daily
- Weekly
- Monthly
- Quarterly
- Yearly

Thinking about the last 12 months...

How often have you used Academic Research Evidence in your work?

- Daily
- Weekly
- Monthly
- Quarterly
- Yearly

Thinking about the last 12 months...

How often have you used Information Collected Online in your work?

- Daily
- Weekly
- Montly
- Quarterly
- Yearly

Broadly, there are three main ways people use information in their work:

Use 1
To act on the information/evidence in specific and direct ways: i.e. often documented use. E.g. to inform development or review of a document such as a project plan, policy, business case, executive brief; to inform writing of project reports, or other reports, or presentations etc.

Use 2
To support, or argue, for certain positions or plans of action: i.e. usually not documented but for a specific use. E.g. using information to influence in meetings and committees, or to influence discussion and decision making in your team, or with your manager/staff, or with internal and external stakeholders, etc.

Use 3
To inform, indirectly or generally, your understanding of an issue; i.e. usually not documented, and not for a specific use. E.g. to understand more about the content area you are working in, etc.

Still thinking about the last 12 months...

How did you use Internal Data and Reports in your work?

Please **rank the three uses** of information by dragging and dropping; from main use of Policy and Legislation (rank as 1) to least (rank as 3).

Please note: To keep the ranking as is (i.e keep 1,2,3 as you see it), you will still need to drag and drop (i.e. move it out of order then back again).

Act on the information/evidence in specific and direct ways (documented use)

Inform, generally or indirectly, understanding of an issue (not documented, not for a specific use)

Support, or argue, for certain positions or plans of action (not documented, but for a specific use)

Still thinking about the last 12 months...

How did you use Policy, Legislation and Legal Information in your work?

Please **rank the three uses** of information; from main use of Policy and Legislation (rank as 1) to least (rank as 3).

Please note: To keep the ranking as is (i.e keep 1,2,3 as you see it), you will still need to drag and drop (i.e. move it out of order then back again).

To act on the information/evidence in specific and direct ways (documented use)

To inform, generally or indirectly, understanding of an issue (not documented, not for a specific purpose)

To support, or argue, for certain positions or plans of action (not documented, but for a specific purpose)

Still thinking about the last 12 months...

How did you use Medical/Clinical Evidence in your work?

Please **rank the three uses** of information; from main use of Medical/Clinical Evidence (rank as 1) to least (rank as 3).

Please note: To keep the ranking as is (i.e keep 1,2,3 as you see it), you will still need to drag and drop (i.e. move it out of order then back again).

To act on the information/evidence in specific and direct ways (documented use)

To inform, generally or indirectly, understanding of an issue (not documented, not for a specific purpose)

To support, or argue, for certain positions or plans of action (not documented, but for a specific purpose)

Still thinking about the last 12 months...

How did you use Experience/Expertise/Advice in your work?

Please **rank the three uses** of information; from main use of Experience/Expertise/Advice (rank as 1) to least (rank as 3).

Please note: To keep the ranking as is (i.e keep 1,2,3 as you see it), you will still need to drag and drop (i.e. move it out of order then back again).

To act on the information/evidence in specific and direct ways (documented use)

To inform, generally or indirectly, understanding of an issue (not documented, not for a specific purpose)

To support, or argue, for certain positions or plans of action (not documented, but for a specific purpose)

Still thinking about the last 12 months...

How did you use Academic Research Evidence in your work?

Please **rank the three uses** of information; from main use of Academic Research Evidence (rank as 1) to least (rank as 3).

Please note: To keep the ranking as is (i.e keep 1,2,3 as you see it), you will still need to drag and drop (i.e. move it out of order then back again).

To act on the information/evidence in specific and direct ways (documented use)

To inform, generally or indirectly, understanding of an issue (not documented, not for a specific purpose)

To support, or argue, for certain positions or plans of action (not documented, but for a specific purpose)

Still thinking about the last 12 months...

How did you use Information Collected Online in your work?

Please **rank the three uses** of information; from main use of Information Collected Online (rank as 1) to least (rank as 3).

Please note: To keep the ranking as is (i.e keep 1,2,3 as you see it), you will still need to drag and drop (i.e. move it out of order then back again).

To act on the information/evidence in specific and direct ways (documented use)

To inform, generally or indirectly, understanding of an issue (not documented, not for a specific purpose)

To support, or argue, for certain positions or plans of action (not documented, but for a specific purpose)

Part 2: Sourcing Information and Evidence

Thinking about the last 12 months...

Who did you speak with to gather Experience/Expertise/Advice to inform your work? ***Please tick as many boxes as relevant***

☐ Your manager

- [ ] Other internal managers
- [ ] Internal subject matter experts (someone in your organisation that is an expert/specialist)
- [ ] Internal colleagues (other people you know who work in your organisation)
- [ ] External colleagues (people you know who work in similar organisations)
- [ ] Employer groups/association members
- [ ] Industry group/association members
- [ ] Health care providers
- [ ] Agents
- [ ] Academics
- [ ] Lawyers
- [ ] Family and friends
- [ ] Consultants
- [ ] Other

Please describe how you gathered Experience/Expertise/Advice to inform your work in the last 12 months?

Where did you obtain the <u>academic research evidence</u> you used in your work in the last 12 months?
*Please **tick** as many **boxes as relevant***

- [ ] Internal library
- [ ] Online/internet
- [ ] Internal peers/experts
- [ ] External peers/experts/stakeholders
- [ ] Public, state or government library/database
- [ ] Academic experts directly
- [ ] University library/database
- [ ] ISCRR - Institute for Safety, Compensation & Recovery Research
- [ ] Consultants
- [ ] Other

Please describe the websites or search engines you have visited online to obtain Academic Research Evidence

Please describe how you obtained Academic Research Evidence from academics directly? E.g. would you phone them, email them, invite them to meet with you etc.

Please describe how you have accessed Academic Research Evidence?

Do you look at the websites of similar organisations nationally and internationally to inform your work?

- never
- Rarely
- Sometimes
- Often
- All of the Time

Thinking about your work in the last 12 months...

Have you...? *Please* ***tick*** *as many* ***boxes as relevant***

- Attended forums/presentations to hear about research findings
- Attended industry forums/presentations
- Invited researchers to give a research perspective in an area of policy development
- Invited stakeholders (unions, employers groups, provider groups, agents) to give an industry perspective in an area of policy development
- Invited researchers to be an active member of a policy development committee
- Regularly used research contacts as a sounding board for policy work
- Contracted a research group or individual to conduct a research review or study?
- Contracted a consultancy company to conduct a research review or study?
- I have not been involved in any of the above

What prompted you to contract a university or academic to conduct research?

What promoted you to contract a consultancy company to conduct a research?

My reason for attending an industry conference/presentation was....

My reason for attending an academic conference/presentation was....

In the last 12 months have you participated in an Academic Research project...?

- Yes
- No

How did you participate in Academic Research? ***Please tick as many boxes as relevant***

- Acted in an advisory capacity to a research team
- Discussed setting of research questions
- Collaborated on development of a competitive research grant
- Active member of a research team
- Collaborated on analysis or writing up of results
- Co-authored research publication
- Assisted in disseminating results

What was the name of the organisation AND/OR academics you worked with on research?

Part 3: Experience of Use of Information

How would you rate your skill level for using the following types of information to inform your work?

	Low	Medium	High	Very High
Internal Data and Reports				
Policy, Legislation and Legal Information				
Medical/Clinical Evidence				
Experience/Expertise/Advice				
Academic Research Evidence				
Information collected online				

How easy or difficult is it for you to access the following types of information for your work?

	Very Difficult	Difficult	Somewhat Difficult	Somewhat Easy	Easy	Very Easy
Internal Data and Reports	○	○	○	○	○	○
Policy, Legislation and Legal Information	○	○	○	○	○	○
Medical/Clinical Evidence	○	○	○	○	○	○
Experience/Expertise/Advice	○	○	○	○	○	○
Academic Research Evidence	○	○	○	○	○	○
Information Collected Online	○	○	○	○	○	○

Do you think there is a need to increase use of the following types of information to inform decision-making in your work?

	Yes	No	In some instances	Not sure
Internal Data and Reports	○	○	○	○
Policy, Legislation and Legal Information	○	○	○	○
Medical/Clinical Evidence	○	○	○	○
Experience/Expertise/Advice	○	○	○	○
Academic Research Evidence	○	○	○	○
Information Collected Online	○	○	○	○

How relevant are the following types of information to your work priorities, issues, focus, etc.?

	Very Relevant	Relevant	Somewhat Relevant	Not Relevant
Internal Data and Reports	○	○	○	○
Policy, Legislation and Legal Information	○	○	○	○
Medical/Clinical Evidence	○	○	○	○
Experience/Expertise/Advice	○	○	○	○
Academic Research Evidence	○	○	○	○
Information Collected Online	○	○	○	○

How easy or difficult is it to use, or apply, Internal Data and Reports to inform your work?

- ○ Very Difficult
- ○ Difficult
- ○ Somewhat Difficult
- ○ Somewhat Easy
- ○ Easy
- ○ Very Easy

How easy or difficult is it to use, or apply, Policy, Legislation and Legal Information to inform your

work?

- Very Difficult
- Difficult
- Somewhat Difficult
- Somewhat Easy
- Easy
- Very Easy

How easy or difficult is it to use, or apply, Medical/Clinical Evidence to inform your work?

- Very Difficult
- Difficult
- Somewhat Difficult
- Somewhat Easy
- Easy
- Very Easy

How easy or difficult is it to use, or apply, Experience/Expertise/Advice to inform your work?

- Very Difficult
- Difficult
- Somewhat Difficult
- Somewhat Easy
- Easy
- Very Easy

How easy or difficult is it to use, or apply, Academic Research Evidence to inform your work?

- Very Difficult
- Difficult
- Somewhat Difficult
- Somewhat Easy
- Easy
- Very Easy

How easy or difficult is it to use, or apply, Information Collected Online to inform your work?

- Very Difficult
- Difficult
- Somewhat Difficult
- Somewhat Easy
- Easy

○ Very Easy

Do you think use of the following types of information is valued in your work?

	Yes	No	In some instances	Not sure
Internal Data and Reports	○	○	○	○
Policy, Legislation and Legal Information	○	○	○	○
Medical/Clinical Evidence	○	○	○	○
Experience/Expertise/Advice	○	○	○	○
Academic Research Evidence	○	○	○	○
Information Collected Online	○	○	○	○

Which of the following barriers to using Academic Research Evidence have you experienced? ***Please tick** as many **boxes as relevant***

- ☐ Lack of time to use research evidence
- ☐ Lack of access to research evidence
- ☐ Research not relevant to needs
- ☐ Research delivery not available in time to address needs
- ☐ Poor verbal communication of research findings
- ☐ Poor visual presentation of research findings
- ☐ Lack of training in how to assess research evidence
- ☐ Workplace culture unsupportive of using academic research
- ☐ Competing interests affecting capacity to use academic research
- ☐ Lack of interaction or collaboration with researchers on a project
- ☐ Lack of actionable messages/recommendations in written reports and summaries
- ☐ Lack of clear summary of research findings
- ☐ x I have never experienced these barriers to use of academic research evidence

Which of the following facilitators to use of Academic Research Evidence have you experienced? *Please **tick** as many **boxes as relevant***

- ☐ Training in how to assess the quality of research evidence
- ☐ Clear summaries of research findings
- ☐ Actionable message/recommendations in written reports and summaries
- ☐ Training in where and how to access research evidence
- ☐ Face to face meetings with academics about research planning and progress
- ☐ Research tailored to identified needs
- ☐ Management support for use of academic research evidence
- ☐ Face to face meetings with academics about research findings
- ☐ Networks or relationships with academics or academic institutions/organisations
- ☐ x I have never experienced these facilitators to use of academic research evidence

How do you feel about use of Academic Research Evidence to inform your work?

- Positive
- Neutral
- Negative

How would you like information to be presented or communicated to you? ***Please tick** as many **boxes as relevant***

- Face to face discussion
- Executive summaries, up to 3 pages
- Full written reports/journal articles/etc
- 1 page summaries
- Tool to demonstrate how info can be used
- Verbal & visual presentation
- Video
- Other

Please describe how you would like different types of information to be presented or communicated to you?

Do you believe there will be negative consequences for <u>not</u> using the following types of information in your work?

	Definitely yes	Probably yes	Probably not	Definitely not
Internal Data and Reports				
Policy, Legislation and Legal Information				
Medical/Clinical Evidence				
Experience/Expertise/Advice				
Academic Research Evidence				
Information Collected Online				

Of the following types of information , which do you intend to use to inform your work in the next 12 months? ***Please tick** as many **boxes as relevant***

- Internal Data and Reports
- Policy, Legislation and Legal Information
- Medical/Clinical Evidence
- Experience/Expertise/Advice
- Academic Research Evidence

☐ Information Collected Online

Are there internal prompts or processes that remind or require you to use the following types of information in your work?

	Yes	No	Not sure
Internal Data and Reports	○	○	○
Policy, Legislation and Legal Information	○	○	○
Medical/Clinical Evidence	○	○	○
Experience/Expertise/Advice	○	○	○
Academic Research Evidence	○	○	○
Information Collected Online	○	○	○

Please describe the internal prompts or processes that remind you to use Academic Research Evidence

Does management encourage or require you to use the following types of information in your work?

	Regularly	Sometimes	Never
Internal Data and Reports	○	○	○
Policy, Legislation and Legal Information	○	○	○
Medical/Clinical Evidence	○	○	○
Experience/Expertise/Advice	○	○	○
Academic Research Evidence	○	○	○
Information Collected Online	○	○	○

Demographics

We collect demographic information because it is important in analysing, understanding, explaining and describing the survey findings.

Please provide your first name and surname (e.g. Bob Smith)

This will only be used to compare answers if you complete this survey again in the future. This information will be kept completely confidential. Only the researchers leading this project will see this information and cannot, as required by Ethics requirements, share this information with anyone else, including anyone in your organisation, or use it for any other purpose.

In the case that you win the iPad and do not want to be contacted on your work email/phone please provide other contact info below

---

Please indicate whether you work are employed by WorkSafe or the TAC

- WorkSafe
- TAC

Please indicate your role level, as described below

- Non-manager - I do not manage people
- Manager - I manage people
- Senior Manager - I manage managers

Role Focus: *(Please tick the option that is most relevant to your work; i.e. even if you do a little of each please indicate the type of **work that you are mostly involved in** on a day to day basis)*

- Program/Projects - My work mainly involves strategy and program and project planning, management, evaluation, and/or mainly program and projects support, tasks supporting program and projects etc. E.g Project Managers, Project Officers, Evaluation Officers, Business Consultants, Data Analysts, Actuaries, Communications, Marketing etc.
- Policy/Legal - My work mainly involves the development and/or review of policy, or legal inforration. E.g. Policy Officers, Lawyers and other Legal staff, etc.
- Operational - My work mainly involves the implementation or delivery of strategy, policy, programs or projects, or tasks that support policy, programs or project implementation and strategic operations. E.g. TAC claims managers, TAC Claims Support, WorkSafe Inspectors, Technical Specialists, Agent relations, Health Provider relations etc.
- Administration/Assistance - My work mainly involves office and business administration, executive assistance or administrative assistance etc. E.g. Admin Officer, Executive Assistant

---

What area of the business are you from? e.g. HSG, Prevention Strategy, etc.

Please indicate your age

- 18 - 25 yrs
- 26 - 35 yrs
- 36 - 45 yrs
- 46 - 55 yrs
- 56 - 65 yrs
- 65+ yrs

Please indicate your gender

- Male
- Female

- Other

Please indicate the highest level of education completed:

- High school
- Certificate/diploma
- Undergraduate degree
- Post-graduate degree (Masters, PhD, MD)

What area or discipline was your undergraduate or postgraduate degree?

Please briefly describe your professional background? *Please describe the types of roles you have had or types of business/business areas you have worked in*

Please indicate how long you have been employed by WorkSafe /TAC:

- Less than 1 yr
- 1 - 5 yrs
- 6 - 10 yrs
- 11- 15 yrs
- 16 - 20 yrs
- 20+ yrs

Please indicate how long you have been in your current role?

- Less than 1 yr
- 1 - 5 yrs
- 6 - 10 yrs
- 11 - 15 yrs
- 16 - 20 yrs
- 20+ yrs

Please indicate how long you have been in government/public sector roles?

- Less than 1 yr
- 1 - 5 yrs

- 6 - 10 yrs
- 11 - 15 yrs
- 16 - 20 yrs
- 20+ yrs

Thank you so much for taking the time to complete this survey,
Your contribution is important and highly valued

If you have anything at all to add or share, or have any further comments or reflections, please do so below. Thanks!
